# Supplementary material for: Human Amniotic Mesenchymal Stem Cells and Fibroblasts Accelerate Wound Repair of Cystic Fibrosis Epithelium
Source: Life (Basel). 2022 May 19;12(5):756. doi: 10.3390/life12050756 (PMC9144497; doi:10.3390/life12050756)
Supplement: Supplementary file 1 [file life-12-00756-s001.zip › life-1707771-supplementary.pdf]

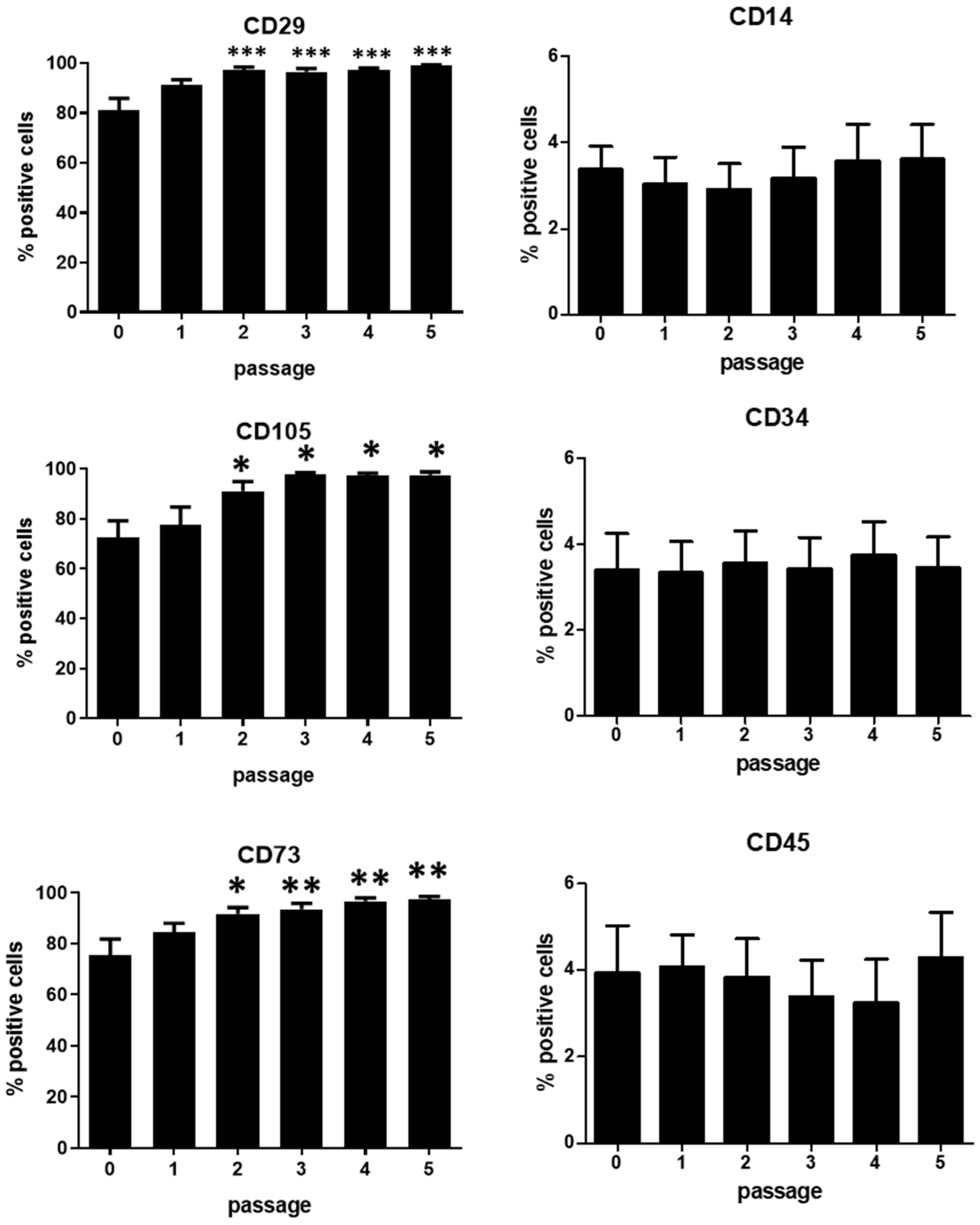

Supplementary Figure S1. Marker expression in hAMSCs. hAMSCs were evaluated for CD29, CD105, CD73, CD14, CD34, and CD 45 at isolation (p0) and p1-5 by flow cytometry. Results are expressed as mean ± SD of triplicates for each isolate. *$p<0.05$; **$p<0.01$, ***$p<0.001$.

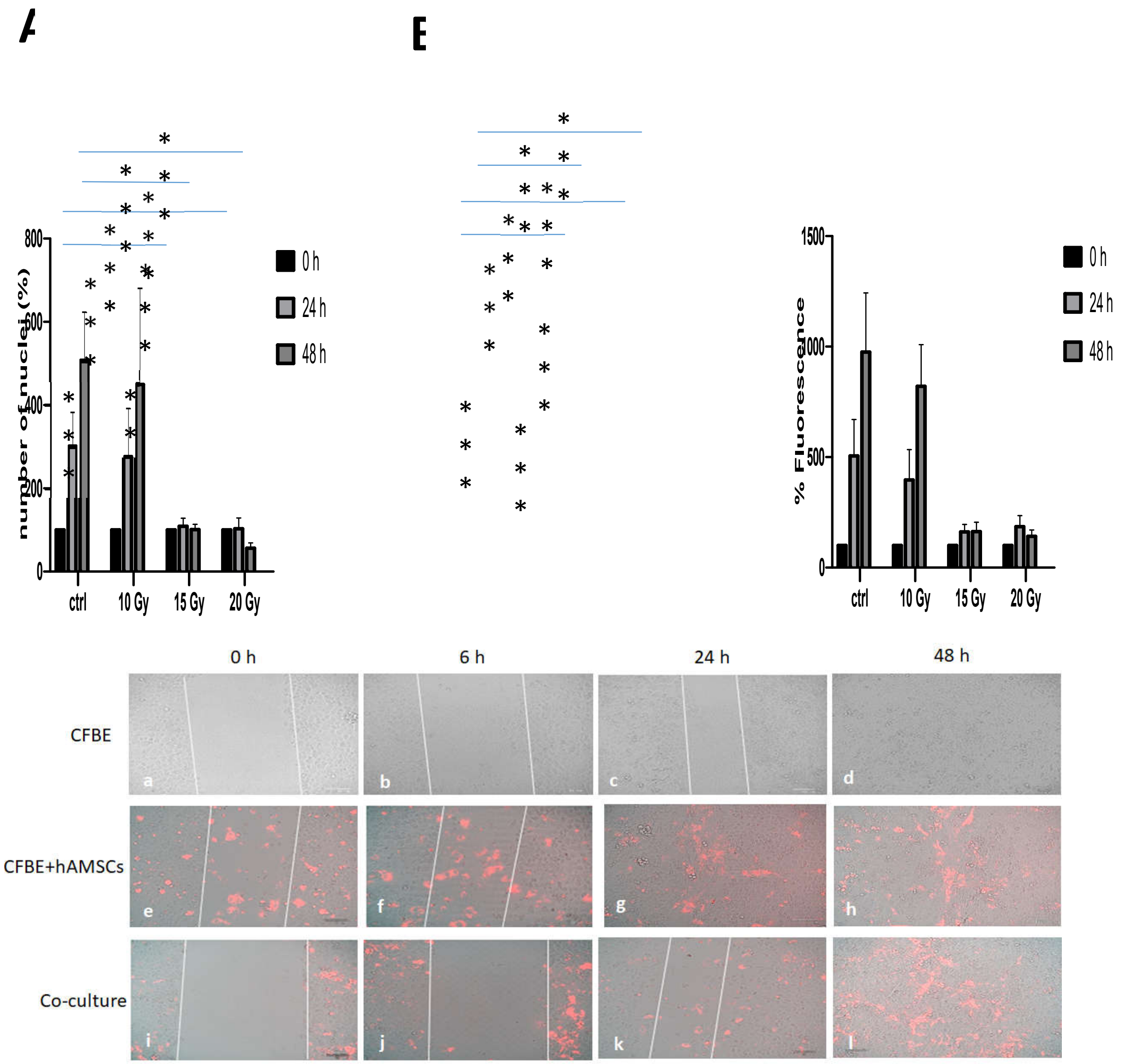

Supplementary Figure S2. Wound closure in the presence of fluorescent hAMSCs. Injury was determined on CFBE (a-d), CFBE with the addition of CM-Dil-labeled hAMSCs (6 x 10^4) after wound (e-h), and CM-Dil-hAMSC:CFBE co-cultures (i-l). Wound closure was evaluated at 0 h (a, e, i), 6 h (b, f, j), 24 h (c, g, k), and 48 h (d, h, l). White lines denote wound edges. Bar = 100 µm.

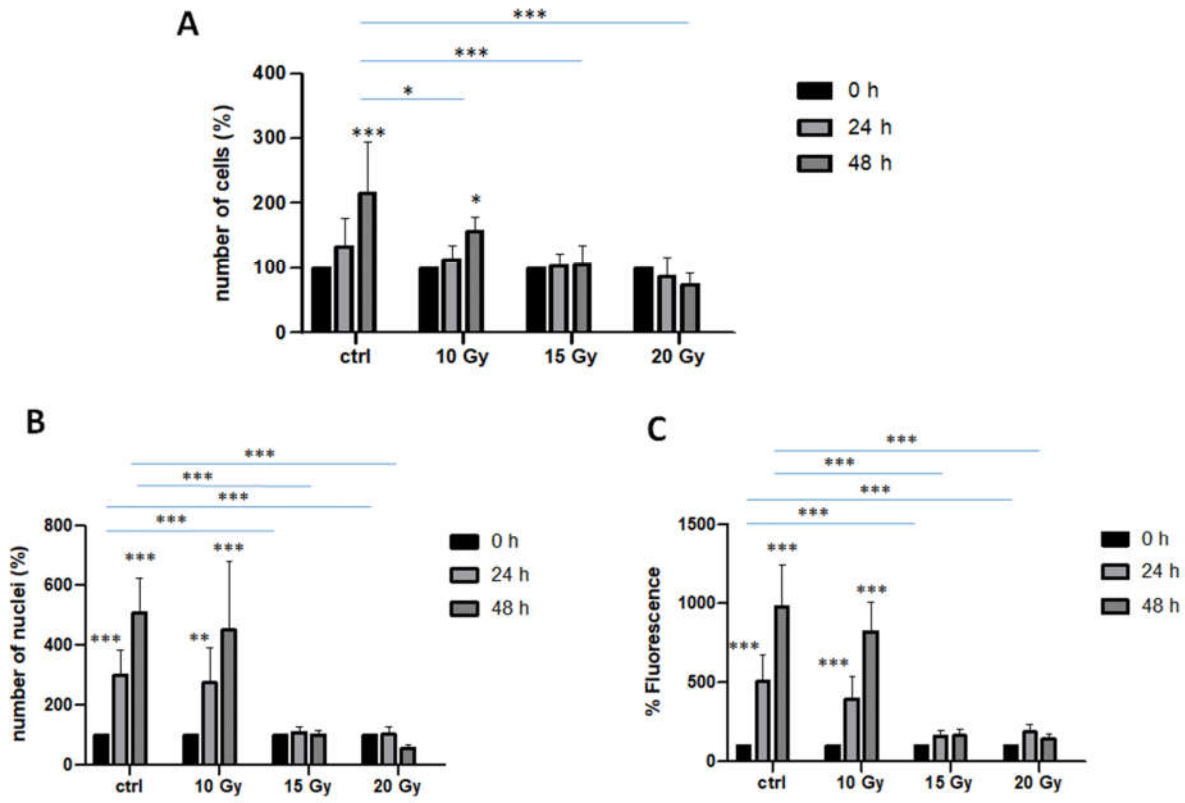

Supplementary Figure S3. CFBE proliferation is blocked by γ-rays. CFBE cells were irradiated at different doses of γ-rays (10, 15, or 20 Gy) and evaluated for cell numbers (A), numbers of DAPI-stained nuclei (B) or DAPI-associated fluorescence (C) as compared with untreated controls (ctrl). In each condition, time 0 is considered as 100%. Results are shown as mean ± SD of three experiments. *p<0.05; **p<0.01; ***p<0.001.

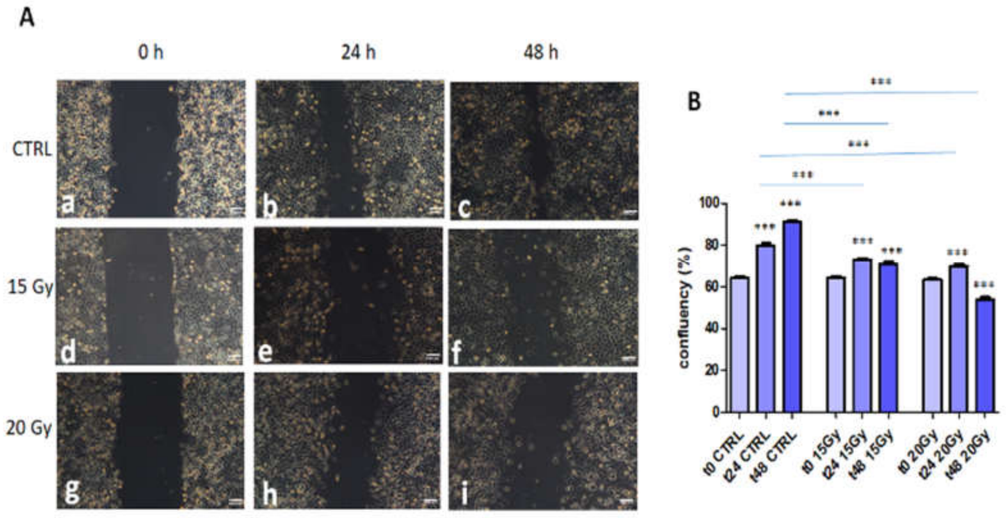

Supplementary Figure S4. Wound closure of CFBE monolayers after γ-irradiation. A) CFBE monolayers were γ-irradiated with 15 Gy (d, e, f) or 20 Gy (g, h, i) and then wounded. Control monolayers were not irradiated (a, b, c). Wound closure was evaluated at time 0 (a, d, g), 24 h (b, e, h) and 48 h (c, f, i). Bar = 100 μm. B) Percentages of wound closure of non-irradiated CFBE, CFBE irradiated with 15 Gy, and CFBE irradiated with 20 Gy. ***p<0.001.
